# Benralizumab Effectiveness in Severe Eosinophilic Asthma with Co-Presence of Bronchiectasis: A Real-World Multicentre Observational Study

**DOI:** 10.3390/jcm12123953

**Published:** 2023-06-09

**Authors:** Raffaele Campisi, Santi Nolasco, Corrado Pelaia, Pietro Impellizzeri, Maria D’Amato, Andrea Portacci, Luisa Ricciardi, Giulia Scioscia, Nunzio Crimi, Nicola Scichilone, Maria Pia Foschino Barbaro, Girolamo Pelaia, Giovanna Elisiana Carpagnano, Alessandro Vatrella, Claudia Crimi

**Affiliations:** 1 Respiratory Medicine Unit, Policlinico “G. Rodolico-San Marco” University Hospital, 95123 Catania, Italy; raffaelemd@hotmail.it (R.C.); nolascos@hotmail.it (S.N.); 2 Department of Clinical and Experimental Medicine, University of Catania, 95123 Catania, Italy; pietroimpellizzeri2018@gmail.com (P.I.); crimi@unict.it (N.C.); 3 Department of Health Sciences, University “Magna Graecia” of Catanzaro, 88100 Catanzaro, Italy; corrado.pelaia@gmail.com (C.P.); pelaia@unicz.it (G.P.); 4 Department of Respiratory Medicine, University “Federico II” of Naples, 80138 Naples, Italy; marielladam@hotmail.it; 5 Department of Translational Biomedicine and Neuroscience, Institute of Respiratory Disease, University “Aldo Moro”, 70121 Bari, Italy; a.portacci01@gmail.com (A.P.); elisiana.carpagnano@uniba.it (G.E.C.); 6 Department of Clinical and Experimental Medicine, University of Messina, 98166 Messina, Italy; luisa.ricciardi@unime.it; 7 Department of Medical and Surgical Sciences, University of Foggia, 71100 Foggia, Italy; giulia.scioscia@unifg.it (G.S.); mariapia.foschino@unifg.it (M.P.F.B.); 8 Division of Respiratory Diseases, Department of Health Promotion Sciences, Maternal and Infant Care, Internal Medicine and Medical Specialties (PROMISE), University of Palermo, 90128 Palermo, Italy; nicola.scichilone@unipa.it; 9 Department of Medicine, Surgery and Dentistry, University of Salerno, 84081 Salerno, Italy; avatrella@unisa.it

**Keywords:** benralizumab, biologics, severe asthma, bronchiectasis

## Abstract

*Introduction*: The co-presence of bronchiectasis (BE) in severe eosinophilic asthma (SEA) is common. Data about the effectiveness of benralizumab in patients with SEA and BE (SEA + BE) are lacking. *Aim*: The aim of this study was to evaluate the effectiveness of benralizumab and remission rates in patients with SEA compared to SEA + BE, also according to BE severity. *Methods*: We conducted a multicentre observational study, including patients with SEA who underwent chest high-resolution computed tomography at baseline. The Bronchiectasis Severity Index (BSI) was used to assess BE severity. Clinical and functional characteristics were collected at baseline and after 6 and 12 months of treatment. *Results*: We included 74 patients with SEA treated with benralizumab, of which 35 (47.2%) showed the co-presence of bronchiectasis (SEA + BE) with a median BSI of 9 (7–11). Overall, benralizumab significantly improved the annual exacerbation rate (*p* < 0.0001), oral corticosteroids (OCS) consumption (*p* < 0.0001) and lung function (*p* < 0.01). After 12 months, significant differences were found between SEA and SEA + BE cohorts in the number of exacerbation-free patients [64.1% vs. 20%, OR 0.14 (95% CI 0.05–0.40), *p* < 0.0001], the proportion of OCS withdrawal [−92.6% vs. −48.6, *p* = 0.0003], and the daily dose of OCS [−5 mg (0 to −12.5) vs. −12.5 mg (−7.5 to −20), *p* = 0.0112]. Remission (zero exacerbations + zero OCS) was achieved more frequently in the SEA cohort [66.7% vs. 14.3%, OR 0.08 (95% CI 0.03–0.27), *p* < 0.0001]. Changes in FEV_1_% and FEF_25–75%_ were inversely correlated with BSI (*r* = −0.36, *p* = 0.0448 and *r* = −0.41, *p* = 0.0191, respectively). *Conclusions*: These data suggest that benralizumab exerts beneficial effects in SEA with or without BE, although the former achieved less OCS sparing and fewer respiratory-function improvements.

## 1. Introduction

Severe asthma is a chronic respiratory disorder with a significant impact on patients’ quality of life due to persistent daily symptoms and frequent exacerbations [1,2]. It is frequently associated with multiple comorbidities, indicating the presence of various underlying immunological processes with distinct endo-phenotypes [3,4,5]. The identification and treatment of these are crucial for improving asthma outcomes [6,7]. An important molecular mechanism of severe asthma is type 2 (T2) inflammation and the presence of T2-high asthma, defined by a blood eosinophil count ≥150 cells/μL and/or FeNO ≥ 20 ppb and/or eosinophils in sputum ≥2%, and/or clinically allergen-driven asthma and/or the need for oral corticosteroids (OCS) [8], which are key features to be evaluated and targeted in the endotyping process.

Eosinophilic inflammation promotes tissue damage and airway remodelling, contributing to the pathogenesis of bronchiectasis (BE), especially in patients with severe eosinophilic asthma (SEA) [9,10,11]. Indeed, the prevalence of BE is significantly higher in patients with SEA (approximately 24–40%) than in those with mild asthma (3%) and their presence is associated with more frequent exacerbations and hospitalisations, higher OCS consumption and poor quality of life [12,13,14]. Thus, SEA with the co-presence of bronchiectasis (SEA + BE) is considered an emerging phenotype with unmet needs [15,16,17].

Benralizumab, a recently approved monoclonal antibody, binds the interleukin (IL)-5 receptor α expressed by eosinophils and basophils and, due to its high affinity for the natural killer cell receptor CD16a, causes rapid depletion of these cells in the blood and airways as a result of antibody-dependent cell-mediated cytotoxicity [18]. Benralizumab has been shown to be effective both in clinical trials and real-life studies in reducing asthma exacerbations and OCS intake while improving pulmonary function and asthma control [19,20,21,22,23,24,25,26,27,28], but its effectiveness in patients with SEA + BE has not yet been investigated.

In this study, we aimed to assess the effectiveness and remission rates of benralizumab treatment in patients with SEA compared to SEA + BE, also according to the severity of bronchiectasis as defined by the Bronchiectasis Severity Index (BSI).

## 2. Materials and Methods

### 2.1. Study Design and Patient Population

We conducted a multicentre, retrospective, observational study on a cohort of patients (≥18 years) with SEA [27] followed-up in these outpatient clinics for severe asthma in Italy, which are part of the “Network for the treatment of severe asthma in Southern Italy”: (1) Respiratory Medicine Unit—A.O.U Policlinico “G. Rodolico—San Marco”, Catania; (2) Pulmonary Unit—A.O.U “Mater Domini”, Catanzaro; (3) Department of Pneumology—A.O. “Dei Colli”, Naples; (4) Respiratory Medicine Unit—A.O.U. Policlinico di Bari “Giovanni XIII”, Bari; (5) Operative Unit of Allergy and Clinical Immunology—Policlinico “G. Martino”, Messina; (6) Institute of Respiratory Diseases—University Hospital of Foggia, Foggia; (7) Respiratory Medicine Unit—A.O.U. “Policlinico Giaccone”, Palermo; (8) Respiratory Medicine Unit—A.O.U. “San Giovanni di Dio and Ruggi d’Aragona”, Salerno.

This study adhered to the Declaration of Helsinki and received approval from the Ethics Committee “Catania 1” at the Policlinico University Hospital (Protocol Number 33/2020/PO) as well as from the local ethics committee of each study site.

### 2.2. Inclusion Criteria

We included patients who fulfilled the following criteria:

1. Diagnosed with SEA as defined by the European Respiratory Society/American Thoracic Society (ERS/ATS) guidelines [29] and compliant to maintenance therapy;
2. Treated with benralizumab (30 mg once every 4 weeks for the first 3 doses, then once every 8 weeks) for at least 12 months between February 2020 and September 2022, with adequate prescription adherence;
3. Underwent high-resolution computed tomography (HRCT) at baseline <6 months before starting the anti-IL-5Rα biologic.

### 2.3. Data Collection and Assessment

The shared data registry of the “Southern Italy Network on Severe Asthma Therapy”, created with the collaboration of all the participating centres, was accessed for data collection. Demographic and clinical characteristics were gathered before biologics initiation (baseline) and at 6 and 12 months of follow-up. Asthma exacerbations were defined as worsening of disease, emergency unit access, hospitalization, and/or use of OCS for ≥3 days or a ≥50% increase in daily OCS dose [30]. Exacerbations treated with cycles of corticosteroids <7 days from each other were considered as the same exacerbation. Exacerbation-free patients were defined based on the absence of asthma exacerbations as defined by guidelines [30]. Chronic mucus hypersecretion (CMH) was defined as cough and excessive production of airway mucus for most days a week for at least three months a year for at least two consecutive years [31]. Patients performed pulmonary function tests according to the ERS/ATS guidelines [32]. Forced vital capacity (FVC), pre-bronchodilator Forced Expiratory Volume in 1 s (FEV_1_%), FEV_1_/FVC ratio, and a mean forced expiratory flow between 25% and 75% of FVC (FEF_25–75_%) were recorded. The best value of three consecutive manoeuvres was expressed as the percentage of the normal value. Levels of asthma control were assessed using the Asthma Control Test (ACT), a short five-point, self-administered scoring system. The total score of the test is 25, with ≥20 indicative of well-controlled asthma, whereas a score ≤19 reflects poor asthma control [33,34]. The fraction of exhaled nitric oxide (FeNO) was performed according to ERS/ATS guidelines [35]. Patients were also assessed to identify who achieved one or more of the composite criteria for clinical remission at 12 months, as defined by the current available definitions in the literature [36,37]: zero exacerbations; zero OCS use; ACT ≥ 20; and pre-bronchodilator FEV_1_ ≥ 80% or an absolute increase ≥100 mL.

### 2.4. Diagnosis and Evaluation of the Severity of Bronchiectasis

All enrolled patients underwent high-resolution computed tomography (HRCT) of the lung, <3 months before starting benralizumab, with 0.5 to 1.5 mm slices. BE diagnosis was made by an expert radiologist based on the lack of bronchial tapering, bronchi visible in the peripheral 10 mm of the costal pleura, and a broncho-arterial ratio >1:1 (or >1.5:1 for more specificity), producing the so-called signet-ring sign [38]. The Bronchiectasis Severity Index (BSI), a multidimensional scoring system which uses a combination of clinical, radiological, and microbiological features, was calculated in patients with SEA + BE. BSI combines age, body mass index (BMI), predicted FEV_1_%, previous hospitalizations in the past two years, number of exacerbations in previous year, modified Medical Research Council (mMRC) dyspnoea score, radiological severity (≥3 lobes involved or presence of cystic BE), presence of *Pseudomonas aeruginosa* and colonization with other organisms. On a score range from 0 to 26, BE were defined as mild (BSI = 0–4 points), moderate (BSI = 5–8 points), or severe (BSI ≥ 9 points) [39].

Microbiology testing was performed on patients’ spontaneous early morning sputum samples and analysed for bacterial, fungal, and mycobacterial cultures. Samples were collected at least on two occasions, with a minimum of three months for one year, to assess the possible presence of chronic colonization [40,41]. Subjects unable to expectorate due to the absence of a productive cough were classified as not having chronic infection for analysis purposes. Patients with a diagnosis of either cystic fibrosis or traction bronchiectasis were excluded.

### 2.5. Statistical Analysis

Continuous data are expressed as mean and standard deviation (±SD), or as median and interquartile range (IQR) for normally and non-normally distributed variables, respectively. Categorical variables are stated as numbers (n) and percentages (%). The normality of data distribution was checked using the Shapiro–Wilk test and the Kolmogorov–Smirnov test. Unpaired Student t-test or Mann–Whitney test were used for comparison of continuous parametric and nonparametric variables at baseline. Fisher exacts or McNemar tests were used for comparisons of categorical variables, when appropriate. Mixed-effect model analysis, with Geisser–Greenhouse correction and Dunnett or Šidák post hoc for repeated measures, were used to compare continuous outcomes at 6 and 12 months with baseline in the entire cohort and to assess treatment response differences between SEA and SEA + BE groups at 6 and 12 months. Odds ratios were also assessed, and 95% confidence intervals (95% CI) were calculated using the Baptista–Pike method. Linear regression analysis, with Spearman’s (*r*) rank correlation coefficients, were developed to evaluate the association between the BSI score and both variables at baseline and changes after 12 months of treatment. Statistical analysis and figures were generated using Prism version 9.4.1 (GraphPad Software Inc., San Diego, CA, USA). A *p* value of < 0.05 (2-sided) was considered statistically significant.

## 3. Results

### 3.1. Baseline Patient Demographics and Clinical Characteristics

**Figure 1:**
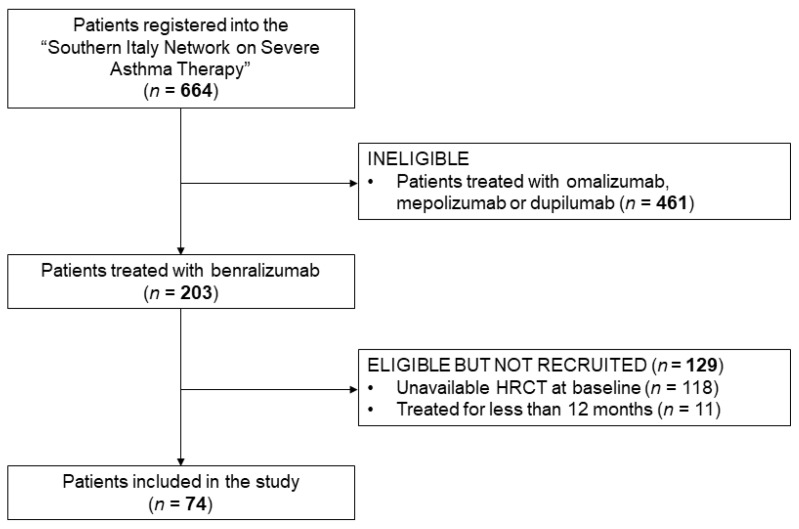
Study flow diagram. Abbreviation: HRCT, high resolution computed tomography.

Among the 664 patients registered in the “Southern Italy Network on Severe Asthma Therapy”, 74 had an HRCT of the chest at baseline and were treated with benralizumab for 12 months. A flow diagram of study participants is shown in Figure 1.

An overview of the study cohort is provided in Table 1.

The mean age was 54.8 ± 11.9 years, including 43 female (58.1%) and 31 men (22.9%), with a median BMI of 25.9 ± 3.8 kg/m^2^. The mean age at SEA onset was 33.8 ± 15.3 years. Median baseline FEV_1_ was 61% (46–77) of the predicted value. All patients were prescribed high-dose inhaled corticosteroids (ICS)—long-acting beta agonists (LABA), with 73% (62 out of 74) requiring maintenance OCS. Four (5.4%) patients were previously treated with anti-IgE (omalizumab) and anti-IL-5 (mepolizumab) therapies. Thirty-five out of 74 patients (47.2%) had the co-presence of BE, as assessed on HRCT, and were included in the SEA + BE group. Noteworthy, 21 of 35 (60%) in the SEA + BE cohort had severe bronchiectasis according to BSI. Statistically significant differences between SEA and SEA + BE patients were observed in the proportion of patients with CMH (51.3% in SEA vs. 82.9% in SEA + BE, *p* = 0.0064), microbial colonization (10.3% in SEA vs. 34.3% in SEA + BE, *p* = 0.0219), asthma exacerbations/year [5 (3.5–7) in SEA vs. 7 (6–16) in SEA + BE, *p* = 0.0012], ACT score [15 (10–18) in SEA vs. 13 (8–16) in SEA + BE, *p* = 0.0175], FEV_1_/FVC% [64% (55.9–76) in SEA vs. 57% (54–65) in SEA + BE, *p* = 0.0202] and in the proportion of patients on OCS (69.2% in SEA vs. 100% in SEA + BE, *p* = 0.0078).

**Figure 2:**
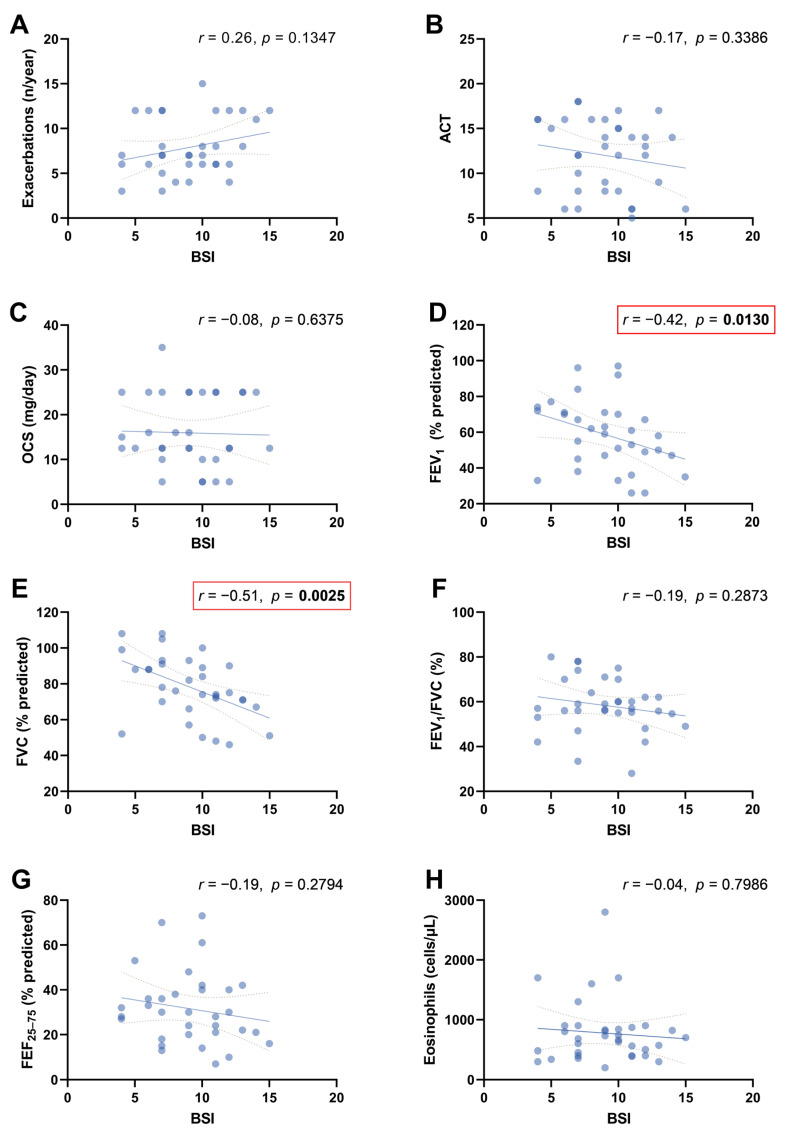
Scatter diagrams and regression lines (95% CI) on correlations between BSI with exacerbations (n/years) (Panel (**A**)); ACT score (Panel (**B**)); OCS (mg/day) (Panel (**C**)); FEV_1_% (Panel (**D**)); FVC% predicted (Panel (**E**)); FEV_1_/FVC% (Panel (**F**)); FEV_25–75_% (Panel (**G**)); and eosinophils (cells/µL) (Panel (**H**)) at baseline. Abbreviations: ACT, Asthma Control Test; FEV_1_, forced expiratory volume in the 1st second; FVC, forced vital capacity; FEF_25–75_%, forced expiratory flow between 25% and 75% of FVC; OCS, oral corticosteroids (prednisone). All parameters are expressed as median values (IQR); *r*: Spearman coefficient.

Considering SEA + BE patients, a significant inverse linear relationship was detected before starting benralizumab between BSI and FEV_1_% (Figure 2D; *r* = −0.42, *p* = 0.0130) and FVC% (Figure 2E; *r* = −0.51, *p* = 0.0025). No significant correlations were found between BSI and the annual exacerbation rate, ACT score, OCS mg/day, FEV_1_/FVC%, FEF_25–75%_, and blood eosinophil count.

### 3.2. Benralizumab Effectiveness in the Entire Cohort

Results are reported in Table 2 and highlight an overall improvement in all examined outcomes.

### 3.3. Effectiveness of Benralizumab in Severe Eosinophilic Asthma with or without Bronchiectasis

Comparative data on benralizumab outcomes in the SEA and SEA + BE populations are summarized in Table 3.

**Figure 3:**
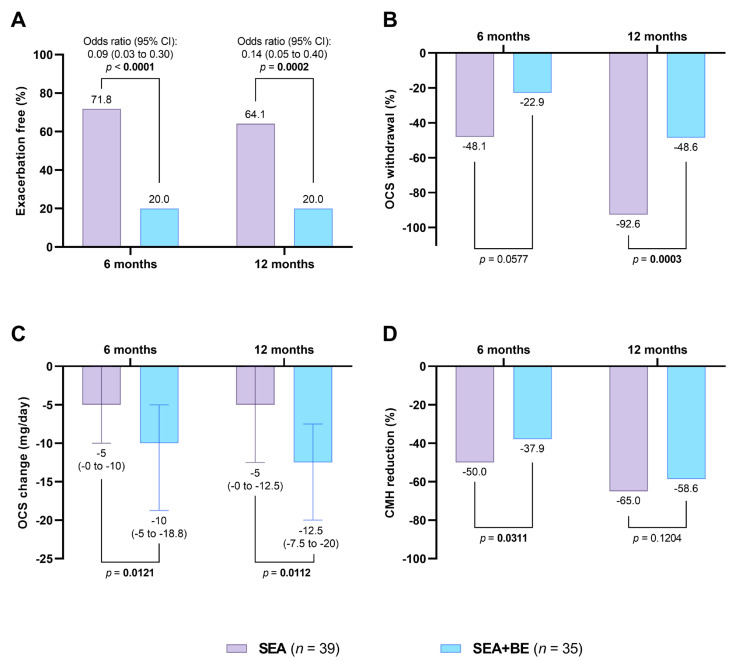
Effects of benralizumab in SEA and SEA + BE groups on the proportion of exacerbation-free patients (Panel (**A**)); OCS withdrawal (Panel (**B**)); OCS dose reduction (mg/day of prednisone) (Panel (**C**)); and CMH reduction (Panel (**D**)). Values are expressed as median (interquartile range [IQR]). Abbreviations: BE, bronchiectasis; CMH, Chronic Mucus Hypersecretion; OCS, oral corticosteroids (prednisone); SEA, severe eosinophilic asthma.

A significant difference in the number of exacerbation-free patients was observed between the two groups after 6 [28 (71.8%) vs. 7 (20%), OR 0.09 (95% CI 0.03–0.30), *p* < 0.0001] and 12 months [25 (64.1%) vs. 7 (20%), OR 0.14 (95% CI 0.05–0.40), *p* < 0.0001] of treatment (Figure 3A). Parallelly, the number of patients on OCS was significantly lower in SEA group in comparison to the SEA + BE cohort [−25 (−92.6%) vs. −17 (−48.6%), respectively, *p* = 0.0003] (Figure 3B) with the daily dose of OCS which was more reduced in the SEA group [−5 mg (0 to −12.5) vs. −12.5 mg (−7.5 to −20), *p* = 0.0112] (Figure 3C). CMH significantly decreased in both the SEA and SEA + BE groups, with a statistically significant difference in favour of the first (−50% vs. −37.9%, *p* = 0.0311), Figure 3D. No statistically significant differences were observed between SEA and SEA + BE patients after 6 and 12 month in terms of the annual exacerbation rate, ACT score and pulmonary function.

### 3.4. Remission According to the Presence of Bronchiectasis

The proportion of patients who achieved remission during benralizumab treatment is summarised in Table 4. Overall, remission was achieved in 31 patients out of 74 (41.9%) according to the less stringent criteria of zero exacerbations and zero OCS after 12 months of therapy, but a statistically significant difference was shown between SEA (26 out of 39, 66.7%) and SEA + BE (5 out of 35, 14.3%) groups, favouring the first [OR 0.08 (95% CI 0.03–0.27), *p* < 0.0001]. Adding the ACT score ≥ 20 and the FEV_1_% or (L), the overall proportion of patients in remission at an advantage in the group without BE (Table 4).

### 3.5. Benralizumab Effectiveness According to Bronchiectasis Severity

At baseline, SEA + BE patients were further divided into two groups: (1) mild-to-moderate BSI (0–8 points) group [14 patients (40%)]; (2) severe BSI (≥9 points) group [21 patients (60%)], as shown in Table 5.

Females were more prevalent among those with severe BSI [5 (35.7%) vs. 16 (76.2%), respectively, *p* = 0.0332]. The median BSI was 7 (4.8–7) in the mild-to-moderate BSI group vs. 11 (9.5–12) in the severe BSI group (*p* < 0.0001). The FEV_1_ (L) was higher in the mild-to-moderate BSI group compared to the severe BSI group [1.8 L (1.4–2.7) vs. 1.4 L (1.1–1.7), *p* = 0.0480].

**Figure 4:**
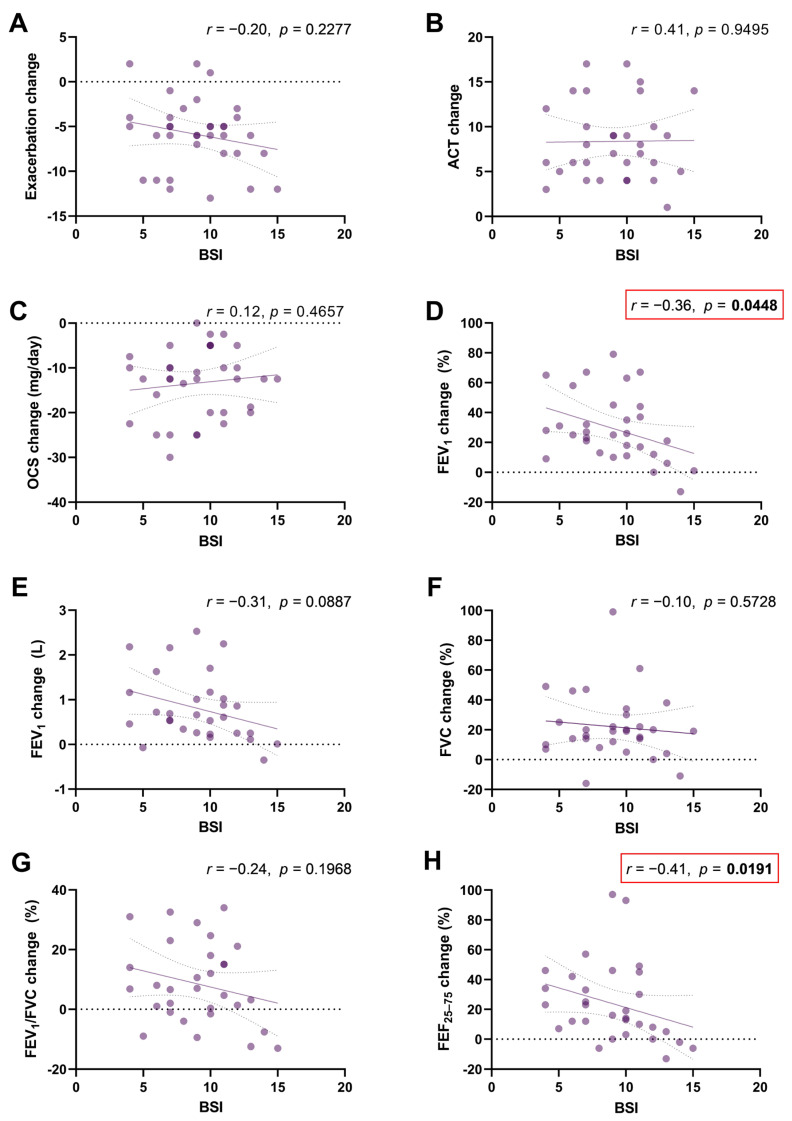
Scatter diagrams and regression lines (95% CI) on correlations between BSI with changes in exacerbations (Panel (**A**)); ACT score (Panel (**B**)); OCS (mg/day) (Panel (**C**)); FEV1% (Panel (**D**)); FEV_1_ (L) (Panel (**E**)); FVC% (Panel (**F**)); FEV_1_/FVC% (Panel (**G**)); and FEV_25–75_% (Panel (**H**)). Abbreviations: ACT, Asthma Control Test; FEV_1_, forced expiratory volume in the 1st second; FVC, forced vital capacity; FEF_25–75_%, forced expiratory flow between 25% and 75% of FVC; OCS, oral corticosteroids (prednisone). All parameters are expressed as median values (IQR); *r*: Spearman coefficient.

We also evaluated how the severity of BE affects the improvement in asthma outcomes during benralizumab treatment. Linear regressions estimated the associations between BSI and outcome changes after treatment (Figure 4). Changes in FEV_1_% and FEF_25–75%_ were inversely correlated with BSI (Figure 4D; r = −0.36, *p* = 0.0448 and Figure 4H; r = −0.41, *p* = 0.0191, respectively). None of the other correlations were statistically significant.

## 4. Discussion

The main finding of this real-world study is that the anti-IL-5Rα biologic treatment determined a significant reduction in asthma exacerbations while improving asthma control and pulmonary function after 6 and 12 months of treatment, in patients with SEA with or without BE. To the best of our knowledge, this is the first study evaluating the effectiveness of benralizumab in SEA patients with the co-presence of BE.

In our cohort of SEA patients, a high prevalence of BE (47.2%) was reported, in line with the current literature [42,43,44]. SEA + BE phenotype is often characterised by poor quality of life and high OCS consumption [12,13,14,45]. Indeed, despite benralizumab leading to an overall positive response, the SEA + BE subgroup maintained a higher proportion of exacerbations and OCS dependence. As such, the odds of achieving remission were 12 times lower in patients with BE. In addition, we found inverse linear relationships between changes in both FEV_1_ and FEF_25–75_% and BSI, showing that those with the most severe BE are less susceptible to lung function improvements.

Previous studies have demonstrated that mepolizumab effectively improved asthma outcomes in a cohort of SEA + BE patients [46,47]. Similarly, a case series of patients with BE and eosinophilic inflammation emphasized the effectiveness of both mepolizumab and benralizumab in significantly reducing airway obstruction and exacerbations [48]. However, the majority of patients in these studies had predominantly mild-to-moderate BE whereas 60% of the patients in our cohort were classified as severe BE based on the BSI score. These findings support the relationship between bronchiectasis and severe asthma, reinforcing the concept that subjects with SEA + BE, particularly the subgroup with severe BSI, had a complex form of difficult-to-control asthma [49]. Therefore, the presence of BE in SEA patients should always be considered, and HRCT should be a mandatory requirement for a comprehensive assessment of the most-severe asthma patients [17,50].

To date, it is still unclear how SEA and BE affect each other. A potential mechanism may be promoted by chronic high OCS intake, which exposes patients to partial immunodeficiency, increasing the risk of infections, impairment of the muco-ciliary layer, and increased mucus production [17,45]. Recent evidence has also suggested that asthma and BE share common inflammatory pathways; while inflammation in BE has traditionally been considered to be primarily neutrophilic, eosinophils are increasingly recognized as key pathogenetic effectors. Recently, Shoemark et al. [51] showed that approximately 20% of patients with BE had eosinophilic inflammation, even excluding patients with asthma and allergic bronchopulmonary aspergillosis. Using FeNO and blood eosinophil counts >300 cells/μL, Oriano et al. [52], demonstrated that the T2-high endotype was present in 31% of BE patients. These studies highlight the heterogeneity of the inflammatory processes in BE, especially in patients with the T2-high phenotype. Patients with SEA + BE have mixed (eosinophilic and neutrophilic) inflammation; therefore, eosinophils also contribute to airways-remodelling phenomena. Eosinophils cause tissue damage through the release of numerous cytotoxic proteins, including the eosinophilic peroxidase and eosinophilic cationic protein, damaging the muco-ciliary epithelium, impairing airways-mucus clearance, and promoting mucus-plug formation [8,9,10,53,54,55,56].

The identification and treatment of comorbidities is crucial for an effective personalized approach to SEA because these patients can be extremely complex and difficult to treat, even with maximal inhaler therapy and the addition of biological drugs.

Our findings support and reinforce the concept that in SEA patients, BE should always be considered as a possible and significant comorbidity. Future research should explore the different efficacies of the available biological therapies in this specific phenotype and also investigate the role of anti-IL-4Rα [57,58] and anti-thymic stromal lymphopoietin (TSLP) [59] monoclonal antibodies, with the goal of determining the best approach for patients with severe uncontrolled asthma with the co-presence of severe bronchiectasis. In particular, the anti-IL-4Rα biologic dupilumab could provide positive outcomes, acting synergistically on eosinophil-homing processes and mucus hypersecretion by blocking IL-13 and IL-4 receptors [58], while the anti-TSLP tezepelumab, by blocking this epithelial “alarmin”, regulates the inflammatory processes in both T2-high and T2-low cascades, acting on the mixed neutrophilic–eosinophilic inflammation hallmark of the SEA + BE phenotype [59].

The strengths of the study include the multicentre design, with patients collected from seven dedicated severe asthma outpatient clinic across Italy, which are part of the “Southern Italy Network on Severe Asthma Therapy” that share a common clinical systematic approach for the management of patients with SEA.

Our study has limitations. First, the small sample size and the retrospective design do not allow for the ruling out of possible unmeasured confounding factors, which could reduce the strength of our results. Second, the observation period was limited to one year, so we cannot tell whether these results will persist in the long term. Third, because of the retrospective observational design of the study, we did not collect information on sputum-sample cytology or data on additional inflammatory blood biomarkers that could have better characterized the nature of the acute exacerbations in the SEA + BE cohort.

In conclusion, our results advance the body of evidence in favour of targeted therapies against eosinophils in the context of T2-high asthma with comorbid BE [60]. Benralizumab led to a depletion in the blood eosinophilic count and improvements in asthma outcomes, including reduced OCS intake and CMH, asthma control, and lung function, which contributed towards the achievement of clinical remission in a significant group of patients, who were able to achieve remission while being completely OCS- and exacerbation-free without a worsening in asthma control. However, as the severity of the bronchiectasis increased, the positive effects of benralizumab diminished, probably because in these patients, although the eosinophilic inflammation was controlled, the neutrophilic component was not dampened.

## Figures and Tables

**Table 1:** Patients baseline demographic and clinical characteristics of severe eosinophilic asthma cohort with or without bronchiectasis.

	All (*n* = 74)	SEA (*n* = 39)	SEA + BE (*n* = 35)	*p*-Value
Age, years, mean (SD)	54.8 (11.9)	53.9 (12.2)	55.8 (11.8)	0.4980
Female, *n* (%)	43 (58.1)	22 (56.4)	21 (60)	0.8161
BMI, mean (SD)	25.9 (3.8)	25.6 (4.2)	26.2 (3.3)	0.4737
Age at onset, years, mean (SD)	33.8 (15.3)	35.8 (16.2)	31.7 (14.1)	0.2520
Patients with positive Skin Prick Tests, *n* (%)	36 (48.6)	19 (48.7)	17 (48.6)	0.9999
**Smoking status**				
Smoking history, *n* (%)	17 (23)	6 (15.4)	11 (31.4)	0.1655
Current smoker, *n* (%)	6 (8.1)	4 (10.3)	2 (5.7)	0.6771
**Comorbidities**				
Patients with GERD, *n* (%)	34 (45.9)	16 (41)	18 (51.4)	0.4940
Patients with CRSwNP, *n* (%)	37 (50)	15 (38.5)	22 (62.9)	0.0618
Patients with BE, *n* (%)	35 (47.3)	0 (0)	35 (100)	n/a
**Bronchiectasis assessment**				
BSI, median (IQR)	9 (7–11)	n/a	9 (7–11)	n/a
Mild BSI (≤4), *n* (%)	3 (4)	n/a	3 (8.6)	n/a
Moderate BSI (5–8), *n* (%)	11 (14.9)	n/a	11 (31.4)	n/a
Severe BSI (≥9), *n* (%)	21 (28.4)	n/a	21 (60)	n/a
Patients with chronic mucus hypersecretion	49 (66.2)	20 (51.3)	29 (82.9)	**0.0064**
Patients with microbial colonization, n (%)	16 (21.6)	4 (10.3)	12 (34.3)	**0.0219**
*P. Aeruginosa*, *n* (%)	4 (5.4)	0 (0)	4 (11.4)	**0.0455**
*A. Fumigatus*, *n* (%)	4 (5.4)	0 (0)	4 (11.4)	**0.0455**
*S. Aureus*, *n* (%)	5 (6.8)	3 (7.7)	2 (5.7)	0.9999
Other, *n* (%)	3 (4)	1 (2.6)	2 (5.7)	0.5999
**Asthma outcomes**				
Asthma exacerbations/year, median (IQR)	6 (4–8)	5 (3.5–7)	7 (6–12)	**0.0012**
ACT, median (IQR)	14 (9–17)	15 (10–18)	13 (8–16)	**0.0175**
FEV_1_, %, median (IQR)	61 (46–77)	67.5 (45.8–84.3)	59 (46–71)	0.2032
FEV_1_, L, median (IQR)	1.7 (1.2–2.3)	2.0 (1.1–2.7)	1.6 (1.2–2.0)	0.1119
FVC, %, median (IQR)	78 (64–93)	83 (60.8–99)	76 (68–90)	0.8298
FEV_1_/FVC, %, median (IQR)	60 (55–71)	64 (55.9–76)	57 (54–65)	**0.0202**
FEF_25–75_, %, median (IQR)	30 (21–42)	32 (22–52)	30 (20–40)	0.1501
**Pharmacologic therapies**				
High dose ICS-LABA, *n* (%)	74 (100)	39 (100)	35 (100)	0.9999
LAMA, *n* (%)	64 (86.5)	31 (79.5)	33 (94.3)	0.0905
Previous anti-IgE/anti IL-5 mAbs, *n* (%)	4 (5.4)	1 (2.6)	3 (8.6)	0.3388
Patients on OCS, *n*, (%)	62 (73)	27 (69.2)	35 (100)	**0.0078**
OCS, mg/day, median (IQR)	12.5 (5–18.3)	5 (0–12.5)	12.5 (10–25)	**0.0001**
**Biomarkers**	54.8 (11.9)	53.9 (12.2)	55.8 (11.8)	0.4980
Eosinophil counts in peripheral blood, cells/μL median (IQR)	630 (435–835)	550 (450–777)	680 (400–870)	0.4800
IgE, UI/mL, median (IQR)	152 (66–465)	158 (54–650)	151 (72.3–387)	0.8465
FeNO, ppb, median (IQR)	47 (35–63)	43 (33–63)	51 (38–66)	0.0678

Abbreviations: BMI, Body Mass Index; GERD, gastro-esophageal reflux; CRSwNP, chronic rhinosinusitis with nasal polyps; BE, bronchiectasis; BSI, Bronchiectasis Severity Index; ACT, Asthma Control Test; OCS, oral corticosteroids (prednisone); ICS-LABA, inhaled corticosteroids-long-acting beta-agonist; LAMA, long-acting muscarinic antagonist; mAbs, monoclonal antibodies; FEV_1_, forced expiratory volume in the 1st second; FVC, forced vital capacity; FEF_25–75_, forced expiratory flow between 25% and 75% of FVC; IgE, immunoglobulin-E; FeNO, fractional exhaled nitric oxide; SEA, severe eosinophilic asthma. Unpaired Student *t*-test or Mann–Whitney test were used for comparison of continuous parametric and nonparametric variables. Fisher exacts test was used for comparisons of categorical variables. Statistically significant *p*-values are highlighted in bold.

**Table 2:** Outcomes after 6 and 12 months of treatment with benralizumab in the whole population.

Total (*n* = 74)	Baseline	6 Months	*p*-Value	12 Months	*p*-Value
**Asthma outcomes**					
Annual exacerbation rate, median (IQR)	6 (4–8)	n/a	n/a	1 (0–2)	**<0.0001**
Exacerbation-free, *n* (%)	n/a	35 (47.3)	n/a	32 (43.2)	n/a
ACT, median (IQR)	14 (9–17)	20 (18–21)	**<0.0001**	22 (20–24)	**<0.0001**
FEV_1_, %, median (IQR)	61 (46–77)	79 (63–95.5)	**<0.0001**	92.5 (67.3–107)	**<0.0001**
FEV_1_, L, median (IQR)	1.7 (1.2–2.3)	2.1 (1.4–2.9)	**<0.0001**	2.5 (1.8–3.4)	**<0.0001**
FVC, %, median (IQR)	78 (64–93)	93 (80–104)	**<0.0001**	98 (83–112)	**<0.0001**
FEV_1_/FVC, %, median (IQR)	60 (55–71)	67 (60–76)	**0.0038**	70 (62–78)	**0.0002**
FEF_25–75_, %, median (IQR)	30 (21–42)	41.5 (30.8–62)	**<0.0001**	57 (35.3–73)	**<0.0001**
**Pharmacologic therapies**					
Patients on OCS, *n*, (%)	62 (83.8)	41 (55.4)	**<0.0001**	20 (27)	**<0.0001**
OCS, mg/day, median (IQR)	12.5 (5–18.3)	2.5 (0–5)	**<0.0001**	0 (0–2.5)	**<0.0001**
**Biomarkers**					
Eosinophil counts in peripheral blood, cells/μL median (IQR)	630 (435–835)	0 (0–50)	**<0.0001**	0 (0–0.0)	**<0.0001**
Patients with chronic mucus hypersecretion, *n* (%)	49 (66.2)	28 (37.8)	**<0.0001**	19 (25.7)	**<0.0001**

Abbreviations: ACT, Asthma Control Test; OCS, oral corticosteroids (prednisone); FEV_1_, forced expiratory volume in the 1st second; FVC, forced vital capacity; FEF_25–75_, forced expiratory flow between 25% and 75% of FVC. Continuous variables were analyzed with mixed-effect model analysis with Geisser–Greenhouse correction and Dunnett or Šidák post hoc for repeated measures. The McNemar test was used for categorical variables. Statistically significant *p*-values are highlighted in bold.

**Table 3:** Comparative data on benralizumab outcomes after 6 and 12 months between severe eosinophilic asthma with or without bronchiectasis.

	6 Months			12 Months		
Total (*n* = 74)	SEA (*n* = 39)	SEA + BE (*n* = 35)	*p*-Value	SEA (*n* = 39)	SEA + BE (*n* = 35)	*p*-Value
**Asthma outcomes**						
Annual exacerbation rate, change from baseline, median (IQR)	n/a	n/a	n/a	−5 (−6 to −3)	−6 (−8 to −4)	0.1308
Exacerbation-free, *n*, (%)	28 (71.8)	7 (20)	**<0.0001**	25 (64.1)	7 (20)	**0.0002**
ACT, change from baseline, median (IQR)	+5 (+2 to +7)	+6 (+3 to +10)	0.3637	+7 (+3 to +13)	+8 (+5 to +12)	0.6022
ACT MCID, *n*, (%)	29 (74.4)	31 (88.6)	0.1458	31 (79.5)	33 (94.3)	0.0905
FEV_1_, %, change from baseline, median (IQR)	+10 (+0.8 to +25)	+12 (+4 to +26)	0.9672	+20 (+3 to +32)	+25 (+12 to +44)	0.2055
FEV_1_, L, change from baseline, median (IQR)	+0.25 (+0.05 to +0.8)	+0.23 (+0.02 to +0.59)	0.8972	+0.65 (+0.24 to +0.98)	+0.61 (+0.25 to +1.16)	0.6662
FVC, %, change from baseline, median (IQR)	+7 (−0.3 to +17)	+10 (+2 to +20)	0.4447	+12 (+2 to +28)	+19 (+10 to +30)	0.4023
FEV_1_/FVC, %, change from baseline, median (IQR)	+6 (0 to +12)	+5.4 (−3.6 to +13.4)	0.9822	+6.8 (−1.9 to +15.6)	+6.8 (−1 to +18)	0.5599
FEF_25–75_, %, change from baseline, median (IQR)	+14.5 (+1.3 to +28.8)	+7 (0 to +13)	0.0811	+19.5 (+6 to +44)	+16 (+5 to +42)	0.9010
**Pharmacologic therapies**						
Patients on OCS, *n*, (%)	−13 (−48.1)	−8 (−22.9)	0.0577	−25 (−92.6)	−17 (−48.6)	**0.0003**
OCS, mg/day, change from baseline, median (IQR)	−5 (0 to −10)	−10 (−5 to −18.8)	**0.0121**	−5 (0 to −12.5)	−12.5 (−7.5 to −20)	**0.0112**
**Biomarkers**						
Eosinophil counts in peripheral blood, cells/μL median (IQR)	0 (0–0.0)	0 (0–50)	0.9999	0 (0–0.0)	0 (0–0.0)	0.9999
Patients with chronic mucus hypersecretion, *n* (%)	−10 (−50)	−11 (−37.9)	**0.0311**	−13 (−65)	−17 (−58.6)	0.1204

Abbreviations: ACT, Asthma Control Test; BE, bronchiectasis; OCS, oral corticosteroids (prednisone); FEV_1_, forced expiratory volume in the 1st second; FVC, forced vital capacity; FEF_25–75_, forced expiratory flow between 25% and 75% of FVC; SEA, severe eosinophilic asthma. Continuous variables were analyzed with mixed-effect model analysis with Geisser–Greenhouse correction and Dunnett or Šidák post hoc for repeated measures. The Fisher exact test was used for categorical variables. Statistically significant *p*-values are highlighted in bold.

**Table 4:** Remission rates after 12 months of benralizumab treatment.

Remission Criteria	Overall (*n* = 74)	SEA (*n* = 39)	SEA + BE (*n* = 35)	OR (95% CI)	*p*-Value
Zero exacerbations + zero OCS, *n* (%)	31 (41.9)	26 (66.7)	5 (14.3)	0.08 (0.03–0.27)	**<0.0001**
Zero exacerbations + zero OCS + ACT ≥ 20, *n* (%)	30 (40.5)	25 (64.1)	5 (14.3)	0.09 (0.03–0.30)	**<0.0001**
Zero exacerbations + zero OCS + ACT ≥ 20 + FEV_1_ ≥ 80%, *n* (%)	27 (36.5)	23 (59)	4 (11.4)	0.09 (0.03–0.31)	**<0.0001**
Zero exacerbations + zero OCS + ACT ≥ 20 + FEV_1_ +100 mL, *n* (%)	23 (31.1)	19 (48.7)	4 (11.4)	0.13 (0.05–0.50)	**0.0009**

Abbreviations: ACT, Asthma Control Test; BE, bronchiectasis; OCS, oral corticosteroids (prednisone); FEV_1_, forced expiratory volume in the 1st second, SEA, severe eosinophilic asthma. Odds ratios 95% confidence intervals (95% CI) were calculated using the Baptista-Pike method. Statistically significant *p*-values are highlighted in bold.

**Table 5:** Comparison at baseline between severe eosinophilic asthma with or without bronchiectasis according to BSI severity.

	SEA + BE (*n* = 35)	Mild-to-Moderate BSI (*n* = 14)	Severe BSI (*n* = 21)	*p*-Value
Age, years, mean (SD)	55.8 (11.8)	56 (13.2)	55.6 (11)	0.9269
Female, *n* (%)	21 (60)	5 (35.7)	16 (76.2)	**0.0332**
BMI, mean (SD)	26.2 (3.3)	26.9 (3.4)	25.7 (3.2)	0.2189
Age at onset, years, mean (SD)	31.7 (14.1)	31.6 (14.3)	31.6 (14.4)	0.8746
Patients with positive Skin Prick Tests, *n* (%)	17 (48.6)	5 (35.7)	12 (57.4)	0.3053
**Smoking status**				
Smoking history, *n* (%)	11 (31.4)	7 (50)	4 (19.1)	0.0725
Current smoker, *n* (%)	2 (5.7)	2 (14.3)	0 (0)	0.1529
**Comorbidities**				
Patients with GERD, *n* (%)	18 (51.4)	7 (50)	11 (52.4)	0.4940
Patients with CRSwNP, *n* (%)	22 (62.9)	11 (78.6)	11 (52.4)	0.1621
Patients with BE, *n* (%)	35 (100)	14 (100)	21 (100)	0.9999
**Bronchiectasis assessment**				
BSI, median (IQR)	9 (7–11)	7 (4.8–7)	11 (9.5–12)	**<0.0001**
Mild BSI (≤4), *n* (%)	3 (8.6)	3 (21.4)	n/a	n/a
Moderate BSI (5–8), *n* (%)	11 (31.4)	11 (78.6)	n/a	n/a
Severe BSI (≥9), *n* (%)	21 (60)	n/a	21 (60)	n/a
Patients with chronic mucus hypersecretion	29 (82.9)	13 (92.9)	16 (76.2)	0.3662
Patients with microbial colonization, *n* (%)	12 (89.7)	3 (21.4)	9 (42.9)	0.2816
*P. Aeruginosa*, *n* (%)	4 (11.4)	1 (7.1)	3 (14.3)	0.1243
*A. Fumigatus*, *n* (%)	4 (11.4)	1 (7.1)	3 (14.3)	0.1243
*S. Aureus*, *n* (%)	2 (5.7)	1 (7.1)	1 (4.8)	0.9999
Other, *n* (%)	2 (5.7)	1 (7.1)	1 (4.8)	0.9999
**Pharmacologic therapies**				
High dose ICS-LABA, *n* (%)	35 (100)	14 (100)	21 (100)	0.9999
LAMA, *n* (%)	33 (94.3)	13 (92.9)	20 (95.3)	0.9999
Previous anti-IgE/anti IL-5 mAbs, *n* (%)	3 (8.6)	1 (7.1)	2 (9.5)	0.9999
Patients on OCS, *n*, (%)	35 (100)	14 (100)	21 (100)	0.9999
OCS, mg/die, median (IQR)	12.5 (10–25)	13.8 (12.5–25)	12.5 (7.5–25)	0.5397
**Asthma outcomes**				
Asthma exacerbations/year, median (IQR)	7 (6–12)	7 (4.7–12)	7 (6–12)	0.9999
ACT, median (IQR)	13 (8–16)	13.5 (8–16)	13 (8–14.5)	0.3698
FEV_1_, %, median (IQR)	59 (46–71)	70 (50–75.5)	52 (38.8–66)	0.0782
FEV_1_, L, median (IQR)	1.6 (1.2–2.0)	1.8 (1.4–2.7)	1.4 (1.1–1.7)	**0.0480**
FVC, %, median (IQR)	76 (68–90)	88 (77–102)	72.5 (59.3–83.5)	0.8298
FEV_1_/FVC, %, median (IQR)	57 (54–65)	58 (51.5–75)	56.7 (54.7–61.5)	0.4725
FEF_25–75_, %, median (IQR)	30 (20–40)	32 (22–37)	26 (20–41.5)	0.6299
**Biomarkers**				
Eosinophil counts in peripheral blood, cells/μL median (IQR)	680 (400–870)	640 (389–1000)	700 (450–835)	0.9537
IgE, UI/mL, median (IQR)	151 (72.3–387)	159 (54–467)	148.5 (71–366)	0.7555
FeNO, ppb, median (IQR)	51 (38–66)	49 (33–63)	52 (37–75)	0.8674

Abbreviations: BMI, Body Mass Index; GERD, gastro-esophageal reflux; CRSwNP, chronic rhinosinusitis with nasal polyps; BE, bronchiectasis; BSI, Bronchiectasis Severity Index; ACT, Asthma Control Test; OCS, oral corticosteroids (prednisone); ICS-LABA, inhaled corticosteroids-long-acting beta-agonist; LAMA, long-acting muscarinic antagonist; mAbs, monoclonal antibodies; FEV_1_, forced expiratory volume in the 1st second; FVC, forced vital capacity; FEF_25–75_, forced expiratory flow between 25% and 75% of FVC; IgE, immunoglobulin-E; FeNO, fractional exhaled nitric oxide; SEA, severe eosinophilic asthma. Unpaired Student t-test or Mann–Whitney test were used for comparison of continuous parametric and nonparametric variables. Fisher exacts test was used for comparisons of categorical variables. Statistically significant *p*-values are highlighted in bold.

## Data Availability

The data presented in this study are available on reasonable request from the corresponding author.
